# Development of an Open-Source Annotated Glaucoma Medication Dataset From Clinical Notes in the Electronic Health Record

**DOI:** 10.1167/tvst.11.11.20

**Published:** 2022-11-28

**Authors:** Jimmy S. Chen, Wei-Chun Lin, Sen Yang, Michael F. Chiang, Michelle R. Hribar

**Affiliations:** 1 Department of Ophthalmology, Casey Eye Institute, Oregon Health & Science University, Portland, OR, USA; 2 Division of Ophthalmology Informatics and Data Science, Viterbi Family Department of Ophthalmology and Shiley Eye Institute, University of California San Diego, La Jolla, CA, USA; 3 Department of Medical Informatics and Clinical Epidemiology, Oregon Health & Science University, Portland, OR, USA; 4 National Eye Institute, National Institutes of Health, Bethesda, MD, USA

**Keywords:** electronic health records, artificial intelligence, big data, glaucoma, medications

## Abstract

**Purpose:**

To describe the methods involved in processing and characteristics of an open dataset of annotated clinical notes from the electronic health record (EHR) annotated for glaucoma medications.

**Methods:**

In this study, 480 clinical notes from office visits, medical record numbers (MRNs), visit identification numbers, provider names, and billing codes were extracted for 480 patients seen for glaucoma by a comprehensive or glaucoma ophthalmologist from January 1, 2019, to August 31, 2020. MRNs and all visit data were de-identified using a hash function with salt from the deidentifyr package. All progress notes were annotated for glaucoma medication name, route, frequency, dosage, and drug use using an open-source annotation tool, Doccano. Annotations were saved separately. All protected health information (PHI) in progress notes and annotated files were de-identified using the published de-identifying algorithm Philter. All progress notes and annotations were manually validated by two ophthalmologists to ensure complete de-identification.

**Results:**

The final dataset contained 5520 annotated sentences, including those with and without medications, for 480 clinical notes. Manual validation revealed 10 instances of remaining PHI which were manually corrected.

**Conclusions:**

Annotated free-text clinical notes can be de-identified for upload as an open dataset. As data availability increases with the adoption of EHRs, free-text open datasets will become increasingly valuable for “big data” research and artificial intelligence development. This dataset is published online and publicly available at https://github.com/jche253/Glaucoma_Med_Dataset.

**Translational Relevance:**

This open access medication dataset may be a source of raw data for future research involving big data and artificial intelligence research using free-text.

## Introduction

Widespread adoption of the electronic health record (EHR) has resulted in significant data available in clinical practice, research, and billing.^1^ These data have proven valuable in part due to their retrospective, longitudinal nature and have stimulated a growing interest in secondary use of EHR data, otherwise known as reuse of available EHR data, in the “big data” movement.^2^^–^^4^ Indeed, large data repositories such as the American Academy of Ophthalmology IRIS Registry^5^^,^^6^ and the National Institutes of Health (NIH) All of US^7^ research program have implemented multicenter collections of structured EHR data, or data entered into specific text fields, as publicly available datasets for big data studies in ophthalmology.

To further support big data research, the National Eye Institute recently published a call for well-annotated and well-documented open datasets, or publicly available data and code, to accelerate innovation in big data analyses and artificial intelligence (AI) algorithm development.^8^ Furthermore, the NIH has previously issued a policy for dataset sharing for all NIH-funded research, though there are no current quality standards for data upload.^9^ Existing open-source datasets have largely been image based, primarily for development of image-based deep learning in ophthalmology.^10^^–^^13^ Recently, Montesano et al.^14^ published the first open dataset of visual field data in hopes of improving glaucoma prediction. Within ophthalmology, there remains a paucity of open-source datasets containing unstructured or free-text data such as clinical notes written during office visits. These notes contain vast amounts of potentially useful data often not documented elsewhere as part of clinical care. However, sharing clinical notes is challenging due to the amount of potential patient protected health information (PHI) as defined by the Health Insurance Portability and Accountability Act (HIPAA) of 1996.^15^

To address this call for open data, we have created an open dataset of clinical visit notes for glaucoma patients for public use with the goals of increasing the amount of open-sourced ophthalmic text data. Glaucoma is a leading cause of irreversible blindness worldwide^16^^,^^17^ and represents an area where big data could potentially improve patient outcomes.^18^ Current studies within predictive AI for glaucoma have focused largely on using large image datasets^19^^–^^23^ and structured EHR data^18^^,^^24^ for glaucoma diagnosis and progression. More recent work by Wang et al.^24^ combined structured EHR and free-text data to improve upon prior models predicting glaucoma prediction. However, the majority of these studies do not use medication data as part of their modeling. Medication management is an important aspect of glaucoma care that is not easily captured because structured medication lists are often incomplete and medication information from free-text clinical notes is difficult to extract. Because notes are often essential for extracting complete medication information for glaucoma patients, we previously developed an automated natural language processing (NLP) model to extract ophthalmic medication entities, including name, frequency, route, and duration, from free-text office visit notes from the EHR that demonstrated high performance on a held-out test set (*F*_1_ scores ranging from 0.75–0.99 for all entities).^25^ We additionally extracted clinically useful data from the notes, such as patient adherence to medications (*F*_1_ = 0.78) and current medication use (*F*_1_ = 0.91), and demonstrated that the model performed well in a proof-of-concept application of medication reconciliation for current medication use between progress notes and medication lists compared to manual reconciliation (*F*_1_ = 0.97).^25^

The purpose of this article is twofold: (1) to describe an open-source dataset consisting of free-text clinical visit notes and their associated active ophthalmic medication information annotations used to train an NLP model, and (2) to describe methods and challenges associated with de-identifying a text-based dataset. This de-identified open-source dataset also addresses the aforementioned key gap in knowledge regarding the need for high-quality text-based open datasets in ophthalmic literature. Our hope is that the publication of the methodologies of this dataset will additionally encourage other researchers to publish their free-text datasets.

## Methods

### Dataset

This study was approved by the Institutional Review Board (IRB) of Oregon Health & Science University and adhered to the tenets of the Declaration of Helsinki. IRB modifications were approved for institutional data sharing. Informed consent was waived for this retrospective study. A sample of free-text visit notes for patients who saw a comprehensive ophthalmologist or glaucoma specialist for glaucoma between January 1, 2019, and August 31, 2020, were extracted from the Oregon Health & Science University EHR clinical data warehouse, Epic (Verona, WI). The patient's medical record number (MRN), visit identification number (VIN), provider, department, age, race, ethnicity, smoking status, and billing code for each accompanying visit were also extracted from this data warehouse. Each visit note in the dataset was reviewed by an author (WCL) and filtered to ensure that prior notes were not completely copied into current notes.

As part of the study, all progress notes were annotated by two authors (WCL, JSC) for ophthalmic medication names (brand, generic, and name abbreviations), route, frequency, dosage, strength, duration, adherence, adverse drug effects, and drug use (i.e., continue or stop) using an open-source annotation tool, Doccano, as previously described in Lin et al.^25^ These medications were annotated by both JSC and WCL in a small cross-validation study, which demonstrated excellent inter-reviewer agreement before proceeding with full annotation independently.^25^ We defined ophthalmic medications as those that were prescribed for either the medical or surgical management of glaucoma and other eye diseases, as well as oral medications (e.g., acetazolamide) and over-the-counter medications such as artificial tears for ocular indications.

### Dataset De-Identification

Demographic and clinical data were also extracted from the EHR data warehouse as part of the query for our clinical notes. These data included the patient's MRN and VIN, as well as the provider, department, billing codes, and demographic features, including age, race/ethnicity, and current smoking status. All demographic data are summarized in Table 1, and all variables except MRN and VIN were removed from this study. The MRN and VIN were then aggregated into a dataframe and removed from the dataset using SHA-256, a cryptogenic hash function, which ultimately generated an encrypted alphanumeric string based on all the extracted features for each patient. To further decrease the risk of decryption, salt (a secret set of characters) was added to the hash function. The resulting unique alphanumeric strings generated for each patient contained no PHI and ultimately served as the filename for each visit note. All de-identification was performed using the deidentifyr package in R (R Foundation for Statistical Computing, Vienna, Austria).^26^

**Table 1.** Demographics of Patients Included in This Study (*N* = 480)

Demographic	Value
Age (y), mean ± SD	72.2 ± 14.6
Race, *n* (%)	
White	393 (81.8)
Black	15 (3.1)
American/Alaska Native	5 (1.0)
Asian	27 (5.6)
Unknown/declined	40 (8.5)
Sex, *n* (%)	
Male	214 (44.6)
Female	266 (55.4)
Ethnicity, *n* (%)	
Hispanic	26 (5.4)
Non-Hispanic	406 (84.6)
Unknown/declined	48 (10.0)
Smoking status, *n* (%)	
Current everyday smoking	15 (3.1)
Current some day smoker	4 (0.8)
Light smoker	2 (0.4)
Former smoker	164 (34.2)
Never smoker	274 (57.1)
Never assessed/unknown	21 (4.4)

The mean age and data for race, sex, ethnicity, and smoking status were extracted for the 480 patients in this dataset.

All annotations and progress notes were then de-identified using a previously published NLP algorithm, Philter,^27^ which was developed to censor PHI in free-text notes. In summary, Philter automatically replaces words thought to be PHI with a series of asterisks, thus preserving word length. Philter was originally trained on data from the i2b2 dataset^28^ and 4500 randomly selected visit notes across all specialties from the University of California at San Francisco. Additionally, Philter uses a whitelist and blacklist to ensure that certain words are always kept or deleted, respectively. Because our progress notes also contained institutional data and PHI that could potentially identify the clinician (e.g., “Casey Eye Institute,” “Director of Glaucoma Service”), we added several terms related to tenure track and institution name that could identify providers to Philter's blacklist. We also added a standardized list of medication names (both generic and brand names) and common ophthalmic abbreviations to Philter's whitelist. The medication list was downloaded from the publicly available ClinCalc DrugSpell Dictionary, which contains medication names from two federal sources: U.S. Food and Drug Administration Orange Book and RxNorm.^29^ The list of common ophthalmic abbreviations was adapted from the EyeGuru “Ophthalmology abbreviations list and ophtho note translator.”^30^

## Results

### Data Summary

Our dataset is publicly available at https://github.com/jche253/Glaucoma_Med_Dataset. Overall, 480 de-identified visit notes from 480 unique patients were included in this dataset. This included 5520 annotated sentences for ophthalmic medications. Additionally, a single de-identified file containing all annotated entities for ophthalmic medications was included. To promote transparency in our data collection methods and intended uses for this data, we have provided a HealthSheet, a structured datasheet specific to healthcare datasets as recommended by Rostamzadeh et al.^31^ based on the original datasheet by Gebru et al.^32^ which was developed for open-datasets for all use cases in AI. This datasheet is provided in the Supplementary Materials. Demographic data for patients included in this dataset are shown in Table 1. This dataset is available under the open-source three-clause Berkeley Source Distribution license.

### Raw Data

The raw dataset is provided as two separate folders:

• Philter_Clinical_Notes—This folder contains all 480 de-identified visit notes, each named with a unique ID and saved as a .txt file. Each .txt file contains a full progress note that was de-identified using Philter. All words containing PHI have been censored with asterisks.
• Philter_Annotations—This folder contains two identical files, one in .txt format and the other in JSONL format. Both files contain complete annotations for all ophthalmic medications mentioned in the progress notes. Each field contains a unique de-identified sentence extracted from a progress note, as well as its accompanying ID and the annotated labels (e.g., name, route, frequency) found in that sentence. The exact location of a given label in the annotated sentence is provided as the position of the starting and ending characters in the given sentence (e.g., “[0, 11, “DRUG”]”).

### Technical Validation

All visit notes and annotations underwent multiple rounds of manual review by two ophthalmologists (JSC, SY) to ensure complete the identification of PHI as defined by HIPAA. Disagreements were resolved by discussion. Overall, 10 instances of remaining PHI were found in the visit notes, of which two were dates with typos (e.g., “1//2019”), seven were names of private clinics, and one was an address. These errors were manually corrected, and upon re-review by both JSC and SY, all 480 notes and the complete annotation file were deemed to be completely de-identified. There were also instances of de-identification of non-PHI (mean ± SD = 6.3 ± 4.7 words per note), which were not manually corrected due to the random nature of these errors. Examples of de-identification of non-PHI and missed PHI are shown in Table 2.

**Table 2.** Examples of Missed Protected Health Information and De-identified Text Not PHI by Philter

Error Type	Original Sentence	De-Identified Sentence	Comments
Missed PHI	HVF 3//2019	HVF *//****	Typo in date was missed by Philter. Original date was changed for privacy. This was manually corrected on subsequent review.
Missed PHI	… currently having heart studies done by NP Cardiology in Alameda clinic	… currently having heart studies done by NP Cardiology in ******* clinic	A clinic name, linked with a potentially identifying medical condition, could be considered PHI. This was manually corrected on subsequent review. Note that an eponym for the original clinic name was used for privacy.
Erroneous de-identification of non-PHI	Pseudoexfoliation glaucoma OU s/p Ahmeds OU	Pseudoexfoliation glaucoma OU s/p ****** OU	Erroneous de-identification of surgical procedure was due to incorrect presumption of identifying a proper noun.
Erroneous de-identification of non-PHI	Reviewed Siri and speak screen on iPhone	Reviewed **** and speak screen on iPhone	Erroneous de-identification of iPhone Siri application that could otherwise be a proper noun.
Erroneous de-identification of non-PHI	Appt notes at time of scheduling: pd *35 copay nDFE	Appt notes at time of scheduling: pd *35 copay ****	Erroneous de-identification of acronym representing “non-dilated fundus exam” that was not in EyeGuru's list of abbreviations.
Erroneous de-identification of non-PHI	Mentions she has been udner lots of stress	Mentions she has been ****lots of stress	Typo in original text was likely thought to be a name by Philter.

Manual reviews of all notes and annotation files were performed to ensure complete de-identification. There were also a number of instances of text not deemed as PHI that were de-identified by Philter.

## Discussion

We present an open-source dataset of de-identified visit notes and its corresponding annotated glaucoma medication information. The data were used to develop an NLP algorithm that extracted medication entities, such as drug name, route, and frequency, with high accuracy for automated medication reconciliation.^25^ Our study fills a gap in knowledge by demonstrating a reproducible methodology for publishing open-source text-based data in ophthalmology.

To date, the majority of published annotated datasets of free-text notes have been focused on de-identification. The largest corpora of publicly available notes from the EHR have focused on annotations of de-identifying PHI and include the i2b2,^28^^,^^33^ MIMIC-III,^34^ and OpenDeID^35^ datasets. These datasets contain 1304 office visit notes across many specialties, visit notes for nearly 40,000 patients admitted to the critical care unit, and 2100 pathology reports, respectively. Other large clinical notes datasets include 3503 visit notes annotated for PHI published by Deleger et al.,^36^ though this dataset is not publicly available, and the TREC Medical Records corpora from 2011 and 2012,^37^ consisting of 93,351 unannotated visit notes across several specialties. Similarly, annotated datasets exist for family history of disease,^38^ words useful in inflammatory bowel disease evaluation,^39^ part-of-speech tagging,^40^ and comprehensive annotation (e.g., anatomy, drugs and dosages, signs and symptoms)^41^; however, none of these datasets is publicly available. Several of these aforementioned datasets have been most notably used for developed de-identification algorithms, particularly for PHI.^27^^,^^33^^,^^42^^–^^44^ Additional use cases for these datasets include big data retrospective analyses for risk factors,^45^^,^^46^ analysis of documentation similarity,^47^ and text extraction for fungal endophthalmitis,^48^ among others. Our dataset complements existing de-identified datasets and offers a new avenue of exploration with annotated ophthalmic medication data.

There are several potential uses for this dataset. First, well-annotated medication data may be used to train and validate more robust, generalizable NLP algorithms to automate medication reconciliation in glaucoma and other ophthalmic diseases in the future. These algorithms have the potential to improve clinician efficiency, improve the quality of patient care, and reduce clinician burnout. Although our previously published NLP model performed well on the extraction of several medication entities, including name, route, frequency, patient adherence, and current medication use,^25^ our model was not trained to extract other clinically useful data such as the duration of medication use (i.e., start and stop date of medications) and adverse effects of medication use. Additionally, although our NLP model achieved excellent performance on a held-out test set, additional validation on external test sets containing notes from other institutions is necessary. Our hope is that our dataset will encourage other researchers to publish similar free-text datasets to accelerate the development of such NLP tools. Second, the annotated dataset may also be used for extraction of other data unavailable in structured or imaging data such as treatment outcomes, medication adherence, and side-effects of therapy. Though our dataset may be slightly limited by errors of over de-identification, the majority of data remain intact. NLP models may be developed using this readily available dataset with additional annotations and combined for use with other existing or future open-source datasets to create generalizable models for various tasks across multiple institutions and specialties. Third, the free-text clinical notes offer opportunities for NLP research beyond data extraction. For example, annotated text may be used to develop algorithms for question answering for both clinically meaningful questions and patient questions (e.g., “are my eyes getting worse?”). In a similar context, algorithms could be developed for summarization of patient data, which may be useful for increasing chart review efficiency in light of the increasing prevalence of note bloat.

One of the most significant challenges to processing and uploading free-text datasets is the inherent variability of documentation and the potential amount of PHI within these data. Our methodology for comprehensive de-identification consisted of two steps: (1) aggregating all potential PHI outside of the notes (i.e., MRNs and VINs) and anonymizing these data using a cryptogenic hash function with added security using salting, and (2) using an automated NLP program, Philter, with institution-specific keywords blacklisted to de-identify our progress notes. We believe this semi-automated methodology is robust and generalizable, with manual review by two graders performed to identify errors in PHI de-identification (Table 2) and ensure complete de-identification. Although manual review is time consuming and may not be scalable for larger datasets, it was a necessary process due to the risk of potentially leaving PHI in our dataset, for which we identified few, but significant, examples of missed PHI. During our manual review, we chose to err on the side of allowing Philter to censor words at risk for being PHI, as opposed to “under-censoring” and leaving identifiable PHI, while focusing on preserving both non-ophthalmic and ophthalmic medication data in our dataset. Although de-identification of these non-PHI elements may affect usage of our dataset for annotation of other entities (such as surgeries performed), we believe that our dataset may still be generally used to train other NLP algorithms, particularly those trained for medication data extraction.

Additionally, there remains a need for NLP de-identification algorithms that generalize across specialties and institutions and achieve high performance beyond those of ideal test conditions. However, achieving de-identification of PHI that minimizes loss of medical information without sacrificing performance in either task may be challenging, as shown in previous work by Baxter et al.^48^ for identification of fungal endophthalmitis in clinical notes. In their study, all instances of the word “Candida,” which otherwise represents a common fungal culprit of endophthalmitis, were censored as proper nouns in the subset of the MIMIC-III dataset used, which may have affected the ability of their NLP methodology to identify fungal infection.^48^ In our study, we also noticed that proper nouns specific to ophthalmic terminology, such as “Ahmed,” were censored by Philter (Table 2). Although we attempted to minimize ophthalmic data lost from these notes by ensuring that common ophthalmic abbreviations were whitelisted by Philter, proper nouns that otherwise could be human names were more often than not de-identified by Philter to minimize risk of re-identification. Because Philter was trained on largely non-ophthalmic clinic notes, this decreased generalization was not completely unexpected. NLP algorithms trained on data from diverse specialties, institutions, and providers would likely increase the performance of de-identification of free-text notes that contain both PHI and medical terminology that would otherwise be mistaken as proper nouns. If training on large-scale datasets is not feasible, other potential solutions include training automated de-identifying algorithms on manually de-identified notes from the institution or specialty of interest to maximize the accuracy of PHI de-identification of a given dataset. Importantly, high-performing de-identification algorithms have the potential to improve the quality of data submitted as part of NIH grant requirements regarding data sharing and to decrease barriers to publishing open data online, including the costs and resources required to process such datasets.

There are several limitations to this dataset and methodology that future work may address. First, our dataset consists of annotated ophthalmic medications for patients treated at a single institution for a specific disease. These clinical notes were written using templates from a limited number of clinicians. It is likely that clinicians at other institutions and departments have different styles of documentation. To address the variability of documentation practices, publication of larger, more diverse datasets will be needed to develop and validate robust, generalizable automated NLP algorithms trained on free-text notes. Second, we only annotated medications directly related to medical and surgical management of ophthalmic diseases, particularly glaucoma. Annotation of all medications and their related entities may be needed to build a comprehensive medication reconciliation tool that could be useful across all specialties. Third, no other structured data from the EHR or imaging were included in this dataset. Although diagnosis and management of glaucoma require a multidimensional approach, the goal of publishing these data was to facilitate extraction of potentially useful data in glaucoma management from free-text. Future work will be needed to evaluate how data extracted from NLP algorithms could guide and improve AI models for glaucoma. For example, future AI models may incorporate both text data, including both structured data and unstructured free-text data such as medications, as well as imaging data.

In conclusion, we have provided an open-source dataset of text-based office visit notes for patients who saw an ophthalmologist for glaucoma, as well as their accompanying annotations of ophthalmic medication entities. Additionally, the methods described in this study may be used as a guide for researchers who wish to publish their free-text data online. Future work may focus on using these data to advance our ability to analyze big data and facilitate development of more robust NLP models for de-identification and data extraction.

## Supplementary Material

Supplement 1
