# Supplementary material for: Development of an Open-Source Annotated Glaucoma Medication Dataset From Clinical Notes in the Electronic Health Record
Source: Transl Vis Sci Technol. 2022 Nov 28;11(11):20. doi: 10.1167/tvst.11.11.20 (PMC9710490; doi:10.1167/tvst.11.11.20)
Supplement: Supplement 1 [file tvst-11-11-20_s001.docx]

**Healthsheet for “Development of An Open-Source Annotated Glaucoma Medication Dataset from Clinical Notes in the Electronic Health Record”**

Jimmy S. Chen, MD; Wei-Chun Lin, MD; Sen Yang, MD; Michael F. Chiang, MD, MA; Michelle R. Hribar, PhD

**

**

If the answer to any of the questions in the questionnaire is N/A, please describe why the answer is N/A (e.g: data not being available)

**Provide a 2 sentence summary of this dataset.**

This dataset consists of clinical notes for glaucoma patients at OHSU seen over 2019. These notes were de-identified for protected health information (PHI) and annotated for glaucoma medications.

**Has the dataset been audited before? If yes, by whom and what are the results?**

No, this dataset has never been previously audited.

**

**

**Version:** A dataset will be considered to have a new version if there are major differences from a previous release. Some examples are a change in the number of patients/participants, or an increase in the data modalities covered.

**Subversion:** A sub-version tends to apply smaller scale changes to a given version. Some datasets in healthcare are released without labels and predefined tasks, or will be later labeled by researchers for specific tasks and problems, to form sub-versions of the dataset.

The following set of questions clarifies the information about the current (latest) version of the dataset. It is important to report the rationale for labeling the data in any of the versions and sub-versions that this datasheet addresses, funding resources, and motivations behind each released version of the dataset.

**Does the dataset get released as static versions or is it dynamically updated?**

a. If static, how many versions of the dataset exist?

b.If dynamic, how frequently is the dataset updated?

This dataset will be static, with updates reserved for errata.

**Is this datasheet created for the original version of the dataset? If not, which version of the dataset is this datasheet for?**

This datasheet was created for the original version of the dataset (1.0).

**Are there any datasheets created for any versions of this dataset?**

No other prior datasheets or prior versions of this dataset exist.

**Does the current version/subversion of the dataset come with predefined task(s), labels, and recommended data splits (e.g., for training, development/validation, testing)?** If yes, please provide a high-level description of the introduced tasks, data splits, and labeling, and explain the rationale behind them. Please provide the related links and references. If not, is there any resource (website, portal, etc.) to keep track of all defined tasks and/or label definitions?

Annotated glaucoma medications are included in this dataset. No splits for training, validation, or testing are included in this dataset.

**If the dataset has multiple versions, and this datasheet represents one of them, answer the following questions:**

a. What are the characteristics that have been changed between different versions of the dataset?

b. Explain the motivation/rationale for creating the current version of the dataset.

c. Does this version have more subjects/patients represented in the data, or fewer?

d. Does this version of the dataset have extended data or new data from the same patients as the older versions? Were any patients, data fields, or data points removed? If so, why?

e. Do we expect more versions of the dataset to be released?

f. Is this datasheet for a sub-version of the dataset? If yes, does this sub-version of the dataset introduce a new task, labeling, and/or recommended data splits? If the answer to any of these questions is yes, explain the rationale behind it.

g. Are you aware of any widespread sub-version(s) of the dataset? If yes, what is the addressed task, or application that is addressed?

N/A, this is the first version (v1.0), and we do not expect further versions to be developed.

**

**

Reasons and motivations behind creating the dataset, including but not limited to funding interests. For any of the following questions, if a healthsheet has already been created for this dataset, then refer to those answers when filling in the below information.

**For what purpose was the dataset created?** Was there a specific task in mind? Was there a specific gap that needed to be filled? Please provide a description.

This dataset was created to address two gaps in knowledge: 1) the lack of open-source text datasets in ophthalmology, and 2) provide annotated medication data that were used to train a previously published NLP algorithm (Lin, et al, TVST, 2021).

**What are the applications that the dataset is meant to address?** (e.g., administrative applications, software applications, research)

This dataset was developed for use in research.

**Are there any types of usage or applications that are discouraged from using this dataset?**

No.

**Who created this dataset (e.g., which team, research group) and on behalf of which entity (e.g., company, institution, organization)?**

**Who funded the creation of the dataset?** If there is an associated grant, please provide the name of the grantor and the grant name and number.

This dataset was created by the ophthalmic informatics research group at Casey Eye Institute, which is the ophthalmology department at Oregon Health & Science University (OHSU). This work was supported by grants R21LM013937 and P30EY10572 from the National Institutes of Health (Bethesda, MD) and by unrestricted departmental funding from Research to Prevent Blindness (New York, NY).

**What is the distribution of backgrounds and experience/expertise of the dataset curators/generators?**

This project was supervised by an informaticist and ophthalmologist both with extensive experience in big data analytics and electronic health records, and was led by an ophthalmology resident with experience in big data research. A key annotator of the dataset was also a trainee in informatics. An incoming ophthalmology resident also participated in validation of the de-identified dataset.

**Any other comments?**

N/A

**

**

**Instances:** Refers to the unit of interest. The unit might be different in the healthsheet compared to the downstream use case: an instance might relate to a patient in the database, but will be used to provide predictions for specific events for that patient, treating each event as separate.

**What do the instances that comprise the dataset represent (e.g., documents, images, people, countries)?** Are there multiple types of instances? Please provide a description

This dataset is composed of text files for both clinical notes and annotations.

**How many instances are there in total (of each type, if appropriate)? (**breakdown based on schema, provide data stats)?

All data is in a text file format. There are 481 text files in the clinical notes portion of the dataset and 1 text file for the annotated portion of the dataset.

**How many patients / subjects does this dataset represent?** Answer this for both the preliminary dataset and the current version of the dataset.

This dataset represents 481 patients.

**Does the dataset contain all possible instances or is it a sample (not necessarily random) of instances from a larger set?** If the dataset is a sample, then what is the larger set? Is the sample representative of the larger set (e.g., geographic coverage)? If so, please describe how this representativeness was validated/verified. If it is not representative of the larger set, please describe why not (e.g., to cover a more diverse range of instances, because instances were withheld or unavailable). Answer this question for the preliminary version and the current version of the dataset in question.

Patients were selected via stratified sampling from all visits to a comprehensive ophthalmologist or glaucoma specialist at OHSU in 2019. Stratified sampling was performed by provider and specialty to ensure that our subset was representative of all providers and the patients they saw.

**What data modality does each patient data consist of?** If the data is hierarchical, provide the modality details for all levels (e.g: text, image, physiological signal). Break down in all levels and specify the modalities and devices.

Each file contains a clinical progress note with PHI text de-identified. The annotated file contains text with the locations of medications in each sentence.

**What data does each instance consist of? “Raw” data (e.g., unprocessed text or images) or features?** In either case, please provide a description.

Processed text as described above.

**Is any information missing from individual instances?** If so, please provide a description, explaining why this information is missing (e.g., because it was unavailable). This does not include intentionally removed information, but might include, e.g., redacted text.

Yes, text was de-identified for PHI as described above.

**Are relationships between individual instances made explicit (e.g., They are all part of the same clinical trial, or a patient has multiple hospital visits and each visit is one instance)?** If so, please describe how these relationships are made explicit.

Only 1 note per patient was included.

**Are there any errors, sources of noise, or redundancies in the dataset?** If so, please provide a description. (e.g., losing data due to battery failure, or in survey data subjects skip the question, radiological sources of noise)

There should not be any errors, though errors in data extraction may be possible.

**Is the dataset self-contained, or does it link to or otherwise rely on external resources (e.g., websites, tweets, other datasets)?** If it links to or relies on external resources:

a. Are there guarantees that they will exist, and remain constant, over time

b. Are there official archival versions of the complete dataset (i.e., including the external resources as they existed at the time the dataset was created)

c. Are there any restrictions (e.g., licenses, fees) associated with any of the external resources that might apply to a future user? Please provide descriptions of all external resources and any restrictions associated with them, as well as links or other access points, as appropriate.

This data is self-contained. It was extracted from the OHSU clinical data warehouse.

**Does the dataset contain data that might be considered confidential (e.g., data that is protected by legal privilege or by doctor-patient confidentiality, data that includes the content of individuals non-public communications)?** If so, please provide a description.

The dataset should no longer contain PHI as a key step in processing our data was de-identification using a natural language processing (NLP) algorithm, Philter, trained to de-identify free-text notes by replacing words concerning for PHI with asterisks. Additional extracted information such as medical record numbers, visit identification numbers, age, race/ethnicity, provider name were de-identified using a cryptogenic hash function with salt.

**Does the dataset contain data that, if viewed directly, might be offensive, insulting, threatening, or might otherwise cause anxiety?** If so, please describe why.

No.

**If the dataset has been de-identified, were any measures taken to avoid the re-identification of individuals?** Examples of such measures: removing patients with rare pathologies or shifting time stamps.

All dates and PHI have been censored using the Philter algorithm as described above. Age < 90 were not altered as defined by the Safe Harbor act.

**Does the dataset contain data that might be considered sensitive in any way (e.g., data that reveals racial or ethnic origins, sexual orientations, religious beliefs, political opinions or union memberships, or locations; financial or health data; biometric or genetic data; forms of government identification, such as social security numbers; criminal history)?** If so, please provide a description.

There is potential for datasets such as free-text clinical notes to contain sensitive data, but upon manual review, there was no text concerning for such.

**

**

**For data that requires a device or equipment for collection or the context of the experiment, answer the following additional questions or provide relevant information based on the device or context that is used (for example)**

a. If there was an MRI machine used, what is the MRI machine and model used?

b. If heart rate was measured what is the device for heart rate variation that is used?

c. If cortisol measurement is reported at multi site, provide details.

d. If smartphones were used to collect the data, provide the names of models. e. Anything else?

N/A

**

**

**Which factors in the data might limit the generalization of potentially derived models?** Is this information available as auxiliary labels for challenge tests? For instance:

a. Number and diversity of devices included in the dataset.

b. Data recording specificities, e.g., the view for a chest x-ray image.

c. Number and diversity of recording sites included in the dataset.

d. Distribution shifts over time

Our dataset was curated at a single institution for patients seen for a single disease.

**What confounding factors might be present in the data?**

a. Interactions between demographic or historically marginalized groups and data recordings, e.g., were women patients recorded in one site, and men in another?

b. Interactions between the labels and data recordings, e.g., were healthy patients recorded on one device and diseased patients on another?

None to our knowledge.

**Any other comments?**

N/A

**

**

**Does the dataset identify any demographic subpopulations (e.g., by age, gender, sex, ethnicity)?** If yes:

a. The reasons that these categories were assessed also should be described in the datasheet.

b. How was this information acquired? Please describe who identified these categories and the source of the classifications used (e.g: self-report or selection, investigator observed, database, electronic health record, survey instrument).

c. If patients’ demographic data is included, are patients aware / did they consent to the collection and use of their demographic information?

d. In some cases, there have been biologically proven associations between demographics and the outcome. Are you aware of similar associations in the tasks covered by this dataset? Should users be wary of specific proxies or associations when using the dataset? If yes, please provide a link to the study, or publication.

e. Is there any mechanism for updating some of this demographic information after its initial collection? For example, if someone wants to change their gender information, what are the mechanisms to do so?

f. Provide a description of the respective distributions of each subgroup population within the dataset.

No, but demographic data is provided as part of the accompanying dataset manuscript.

**If no, is there any regulation that prevents demographic data collection in your study (for example, the country that the data is collected in)?**

None.

**

**

**Was there any pre-processing for the deidentification of the patients?** Provide the answer for the preliminary and the current version of the dataset.

Yes. As above, a key step in processing our data was de-identification using a natural language processing (NLP) algorithm, Philter, trained to de-identify free-text notes by replacing words concerning for PHI with asterisks.

Additional extracted information such as medical record numbers, visit identification numbers, age, race/ethnicity, provider name were de-identified using a cryptogenic hash function with salt.

**Was there any pre-processing for cleaning the data?** Provide the answer for the preliminary and the current version of the dataset.

No.

**Was the “raw” data (post de-identification) saved in addition to the preprocessed/cleaned data (e.g., to support unanticipated future uses)?** If so, please provide a link or other access point to the “raw” data

No, only the final de-identified and cleaned data was provided.

**Were instances excluded from the dataset at the time of preprocessing?** If so, why? For example, instances related to patients under 18 might be discarded.

No.

**If the dataset is a sample from a larger set, what was the sampling strategy (e.g., deterministic, probabilistic with specific sampling probabilities)?** Answer this question for both the preliminary dataset and the current version of the dataset.

As above - stratified sampling was performed for all glaucoma patients seen by a comprehensive or glaucoma ophthalmologist at OHSU in 2019.

**Any other comments?**

N/A

**

**

**Is there an explicit label or target associated with each data instance?** Please respond for both the preliminary dataset and the current version.

a. If yes:

i) What are the labels provided?

ii) Who performed the labeling? For example, was the labeling done by a clinician, ML researcher, university or hospital?

b. What labeling strategy was used?

i) Gold standard label available in the data (e.g., cancers validated by biopsies)

ii) Proxy label computed from available data:

1. Which label definition was used? (e.g., Acute Kidney Injury has multiple definitions)

2. Which tables and features were considered to compute the label?

iii) Which proportion of the data has gold standard labels?

c. Human-labeled data

i) How many labellers were considered?

ii) What is the demographic of the labellers? (countries of residence, of origin, number of years of experience, age, gender, race, ethnicity, . . . )

iii) What guidelines did they follow?

iv) How many labellers provide a label per instance? If multiple labellers per instance:

1. What is the rater agreement? How was disagreement handled?

2. Are all labels provided, or summaries (e.g., maximum vote)?

v) Is there any subjective source of information that may lead to inconsistencies in the responses? (e.g: multiple people answering a survey having different interpretation of scales, multiple clinicians using scores, or notes)

vi) On average, how much time was required to annotate each instance?

vii) Were the raters compensated for their time? If so, by whom and what amount? What was the compensation strategy (e.g., fixed number of cases, compensated per hour, per cases per hour)?

a. Yes.

i) ophthalmic medications were annotated for name, route, frequency, dosage.

ii) An informatics PhD student and ophthalmology resident annotated the dataset.

b. As above

i and ii) No gold standard or proxy labels available

1. N/A

2. N/A

iii. N/A

c.

i. 2 labellers participated as above

ii. Both graders were Asian and in the age range of 25-34

iii. No specific guidelines exist for annotating medications.

iv) Each labeller annotated different sets of notes, together annotating 481 notes.

v) N/A

vi) Each note required around 5-10 minutes.

vii) Raters were not compensated for their time.

**What are the human level performances in the applications that the dataset is supposed to address?**

Currently, human annotations for medications is the gold standard

**Is the software used to preprocess/clean/label the instances available?** If so, please provide a link or other access point.

Doccano is the open-source annotation text tool that was used to annotate the medications in the dataset. The link is below: https://github.com/doccano/doccano.

**Is there any guideline that the future researchers are recommended to follow when creating new labels / defining new tasks?**

There are no predefined guidelines for how to best annotate notes for medications, as well as other entities.

**Are there recommended data splits (e.g., training, development/validation, testing)?** Are there units of data to consider, whatever the task? If so, please provide a description of these splits, explaining the rationale behind them. Please provide the answer for both the preliminary dataset and the current version or any sub-version that is widely used.

None - no predefined splits were included in this dataset.

**Any other comments?**

N/A

**

**

**Were any REB/IRB approval (e.g., by an institutional review board or research ethics board) received?** If so, please provide a description of these review processes, including the outcomes, as well as a link or other access point to any supporting documentation.

This study was approved by the OHSU institutional review board. Due to the retrospective nature of this study consisting of data extraction from the EHR, the need for informed consent was waived.

**How was the data associated with each instance acquired?** Was the data directly observable (e.g., medical images, labs or vitals), reported by subjects (e.g., survey responses, pain levels, itching/burning sensations), or indirectly inferred/derived from other data (e.g., part-of-speech tags, model based guesses for age or language)? If data was reported by subjects or indirectly inferred/derived from other data, was the data validated/verified? If so, please describe how.

Medical record numbers (MRN) visit identification numbers (VIN), age, race/ethnicity, smoking status, provider department, and billing code (ICD-10) were extracted along with each progress note. These data were also extracted from the OHSU EHR data warehouse (Epic; Verona, WI)

**What mechanisms or procedures were used to collect the data (e.g., hardware apparatus or sensor, manual human curation, software program, software API)?** How were these mechanisms or procedures validated? Provide the answer for all modalities and collected data. Has this information been changed through the process? If so, explain why.

N/A

**Who was involved in the data collection process (e.g., patients, clinicians, doctors, ML researchers, hospital staff, vendors, etc) and how were they compensated (e.g., how much were contributors paid)?**

These data were collected as part of routine clinical care. No additional compensation was provided to those providing clinical care to patients .

**Over what timeframe was the data collected? Does this timeframe match the creation timeframe of the data associated with the instances (e.g., recent crawl of old news articles)?** If not, please describe the timeframe in which the data associated with the instances was created.

This data was collected from 1/1/2019 to 12/31/2019, and corresponds to the notes extracted for this study.

**Does the dataset relate to people?** If not, you may skip the remaining questions in this section.

Yes.

**Did you collect the data from the individuals in question directly, or obtain it via third parties or other sources (e.g., hospitals, app company)?**

These data were obtained from a data warehouse containing uploaded data including demographic information and notes, and not directly from patients nor from manual review of their medical records.

**Were the individuals in question notified about the data collection?** If so, please describe (or show with screenshots or other information) how notice was provided, and provide a link or other access point to, or otherwise reproduce, the exact language of the notification itself.

No.

**Did the individuals in question consent to the collection and use of their data?** If so, please describe (or show with screenshots or other information) how consent was requested and provided, and provide a link or other access point to, or otherwise reproduce, the exact language to which the individuals consented.

Informed consent was waived by the institutional review board for this retrospective study.

**If consent was obtained, were the consenting individuals provided with a mechanism to revoke their consent in the future or for certain uses?** If so, please provide a description, as well as a link or other access point to the mechanism (if appropriate).

No.

**In which countries was the data collected?**

United States.

**Has an analysis of the potential impact of the dataset and its use on data subjects (e.g., a data protection impact analysis) been conducted?** If so, please provide a description of this analysis, including the outcomes, as well as a link or other access point to any supporting documentation.

No.

**

**

**Is there any language-based communication with patients?** If yes, describe the choices of language(s) for communication. (for example, if there is an app used for communication, what are the language options?)

No.

**What are the accessibility measurements and what aspects were considered when the study was designed and implemented?**

N/A

**If data is part of a clinical study, what are the inclusion criteria?**

This data was not part of a clinical study, but inclusion criteria included 1 note per patient who saw a comprehensive ophthalmologist or glaucoma specialist in 2019.

**

**

**Has the dataset been used for any tasks already?**

Yes, this data was previously used to train a natural language processing (NLP) model for named entity recognition and extraction of words relevant to ophthalmic medications. The citation for the paper is below:

Wei-Chun Lin, Joel Kaluzny, Jimmy S Chen, Michael F Chiang, Michelle Hribar; Medication List Extraction Using Natural Language Processing for Glaucoma Patients. Invest. Ophthalmol. Vis. Sci. 2021;62(8):1001.

**Does using the dataset require the citation of the paper or any other forms of acknowledgement?** If yes, is it easily accessible through google scholar or other repositories.

Yes, use of the dataset will require citation, which can include both the DOI for the Github repository and the citation for the accompanying dataset published to *Translational Vision Science & Technology*.

**Is there a repository that links to any or all papers or systems that use the dataset?** If so, please provide a link or other access point.

No.

**Is there anything about the composition of the dataset or the way it was collected and preprocessed/cleaned/labeled that might impact future uses?** For example, is there anything that a future user might need to know to avoid uses that could result in unfair treatment of individuals or groups (e.g., stereotyping, quality of service issues) or other undesirable harms (e.g., financial harms, legal risks) If so, please provide a description. Is there anything a future user could do to mitigate these undesirable harms?

No.

**Are there tasks for which the dataset should not be used?** If so, please provide a description. (for example, dataset creators could recommend against using the dataset for considering immigration cases, as part of insurance policies)

No.

**Any other comments?**

N/A

**

**

**Will the dataset be distributed to third parties outside of the entity (e.g., company, institution, organization) on behalf of which the dataset was created?** If so, please provide a description.

This dataset will be made publicly available. All third parties are welcome to use this dataset.

**How will the dataset be distributed (e.g., tarball on website, API, GitHub)?** Does the dataset have a digital object identifier (DOI)?

This data will be uploaded to Github at the following link: https://github.com/jche253/Glaucoma_Med_Dataset. The DOI for this repository will be assigned upon publication to *TVST*.

**When will the dataset be distributed?**

This dataset will be publicly available upon publication of the *TVST* manuscript.

**Assuming the dataset is available, will it be/is the dataset distributed under a copyright or other intellectual property (IP) license, and/or under applicable terms of use (ToU)?** If so, please describe this license and/or ToU, and provide a link or other access point to, or otherwise reproduce, any relevant licensing terms or ToU, as well as any fees associated with these restrictions.

This data will be available under the three-clause Berkeley Source Distribution .

**Have any third parties imposed IP-based or other restrictions on the data associated with the instances?** If so, please describe these restrictions, and provide a link or other access point to, or otherwise reproduce, any relevant licensing terms, as well as any fees associated with these restrictions.

No.

**Do any export controls or other regulatory restrictions apply to the dataset or to individual instances?** If so, please describe these restrictions, and provide a link or other access point to, or otherwise reproduce, any supporting documentation.

No.

**Any other comments?**

N/A

**

**

**Who will be supporting/hosting/maintaining the dataset?**

Jimmy S. Chen, MD will host the dataset indefinitely, and Jimmy S. Chen, MD, Michelle Hribar, PhD and Wei-Chun Lin, MD will both be responsible for maintaining the dataset.

**How can the owner/curator/manager of the dataset be contacted (e.g., email address)?**

Please contact the supervising researcher Michelle Hribar at [hribarm@ohsu.edu](mailto:hribarm@ohsu.edu) with any questions.

**Is there an erratum?** If so, please provide a link or other access point.

No, but will provide errata as necessary.

**Will the dataset be updated (e.g., to correct labeling errors, add new instances, delete instances)?** If so, please describe how often, by whom, and how updates will be communicated to users (e.g., mailing list, GitHub)?

Yes, this dataset will be updated as needed. Updates will be posted to GitHub.

**If the dataset relates to people, are there applicable limits on the retention of the data associated with the instances (e.g., were individuals in question told that their data would be retained for a fixed period of time and then deleted)**? If so, please describe these limits and explain how they will be enforced.

No.

**Will older versions of the dataset continue to be supported/hosted/maintained?** If so, please describe how. If not, please describe how its obsolescence will be communicated to users.

Yes. The dataset will be updated via GitHub, and older versions can be accessed as part of the GitHub functionality. All documented changes will be noted on the GitHub page.

**If others want to extend/augment/build on/contribute to the dataset, is there a mechanism for them to do so?** If so, please provide a description. Will these contributions be validated/verified? If so, please describe how. If not, why not? Is there a process for communicating/distributing these contributions to other users? If so, please provide a description.

Individuals are free to fork this dataset and modify for their own purposes, but must cite and give credit to the original publication.

**Any other comments?**

N/A
